# Relationship between Patient Demographic Characteristics, Valproic Acid Dosage and Clearance in Adult Iranian Patients

**Published:** 2012

**Authors:** Tamara Aghebati, Mohsen Foroughipour, Mahmoud Reza Azarpazhooh, Naghme Mokhber, Mohammad Hasanzadeh Khayat, Naser Vahdati, Amir Hooshang Mohammadpour

**Affiliations:** 1 *Pharmaceutical Research Centre, Mashhad University of Medical Sciences, Mashhad, Iran*; 2 *Department of Neurology, Faculty of Medicine, Ghaem Hospital, Mashhad University of Medical Sciences, Mashhad, Iran*; 3 *Department of Psychiatry, Faculty of Medicine & Psychiatric Research Centre, Ebn-sina Hospital, Mashad University of Medical Sciences, Mashhad, Iran*; 4 *Department of Medicinal Chemistry, School of Pharmacy, Mashhad University of Medical Sciences, Mashhad, Iran*; 5 *Department of Pharmacodinamy & Toxicology, School of Pharmacy, Mashhad University of Medical Sciences, Mashhad, Iran*

**Keywords:** Clearance, Demographic characteristic, Pharmacokinetic, Valproic acid

## Abstract

**Objective(s):**

As there are conflicting findings regarding the clearance-dose and patient characteristics relationships for valproic acid (VPA), this study was conducted to investigate the relationship between patient demographic characteristics, VPA dosage and the drug clearance in adult Iranian patients.

**Materials and Methods:**

Patients (N= 47) were either on monotherapy with VPA or were under co-treatment with drugs that have no effect on VPA pharmacokinetic (PK) profile. All of the patients received VPA at therapeutic dose. Steady state trough plasma concentrations of VPA were determined by Fluorescence Polarization Immunoassay (FPIA) and VPA apparent clearance (CL/F) were calculated in each patient.

**Results:**

Mean VPA dose and VPA CL/F were 8.93±2.2 mg/kg/day and 0.65±0.55 l/hr respectively. No significant correlations were found between VPA CL/F and patients' age, TBW and VPA dose. VPA CL/F values of male and female patients were compared and no significant difference between these two groups was noted (*P*> 0.05). Significant correlation between VPA dose and total trough plasma concentration was found (*P*= 0.001). Mean total VPA plasma concentration was 54.51±23.74 mg/l.

**Conclusion:**

Our study showed PK of VPA was not affected by age, sex, TBW and VPA dose. However, for detailed results and construction of VPA PK model in Iranian patients, it is necessary to evaluate VPA PK in a larger sample size with different VPA doses, age and TBW ranges.

## Introduction

Valproic acid (VPA) possesses favorable properties which bring about its wide use as an anticonvulsive therapy. These properties include efficacy against both partial and generalized seizure, a relatively low potential for side effects and low propensity to cause drug interaction due to its lack of enzyme inducing activity. This drug also has mood stabilizing effect and have established efficacy in management of bipolar disorder (1).

VPA has dose dependent pharmacokinetic (PK) at high doses. In addition to the fact that various factors may affect VPA PK profile, there is a significant inter-individual variation in its PK parameters (2, 3). Furthermore significant variability has been reported in plasma concentration – dose relationship for this drug (3, 4). Because clinical outcomes of VPA are more closely related to drug level than dose, therapeutic drug monitoring (TDM) of this drug would be more rational for clinicians. That makes it possible to prescribe dosage regimen more properly and also to optimize therapeutic efficacy and avoid toxicity and interaction with other drugs ( both antiepileptic drugs (AED_s_) and non-AED_s _) (3, 5).

Although the effects of VPA dose and demographic factors on VPA PK have been frequently studied before, the results are often controversial. Several factors may contribute to these conflicting data including heterogeneous patient populations, intra-patient variation in PK parameters and polytherapy (4). Since there is no study to evaluate VPA PK properties in Iranian adult patients, we conducted present study to evaluate influence of these affecting factors on VPA PK as a PK model in Iranian patients.

## Materials and Methods

***Patients***

This study was approved by the Ethics Committee of MUMS (Mashhad University of Medical Sciences). It was carried out prospectively in the psychiatric and neurologic clinic of Ebn –sina and Ghaem Hospitals of Mashhad University of Medical Science in Iran. Forty seven patients who fulfilled the following inclusion and exclusion criteria entered this study. Inclusion criteria were: 

a) Receiving a constant dose of VPA at least for 5 days to assume steady state. b) Taking VPA either alone or with other drugs that do not affect VPA clearance. 

Exclusion criteria were: 

a) Patients with abnormal renal function tests. 

b) Patients with abnormal liver function tests (elevated hepatic enzymes 3 times more than normal limit). 

c) A history of congestive heart failure, thyroid disorders and diabetes. 

Whenever a blood sample was taken, all relevant demographic data (e.g. age, gender, total body weight (TBW), past medical history and habit history, medication details (time of the last dose administered, sampling time, duration of therapy, concurrent medication and adverse drug reactions) were recorded. In addition several laboratory tests (CBC _diff _, FBS, SCr, BUN, ALT, AST, thyroid function tests) were performed.

***Blood sampling and drug assays***

VPA BID (tablet form) was provided by RUZ DARU (Iran). Serum samples were taken before the administration of the morning dose. Total VPA plasma concentration was measured by fluorescence polarization immunoassay (FPIA) method. TDxFLx® Analyzers supplied by Abbott Diagnostics, IL, USA, were used for the study. Calibrator, control and patient blood samples were transferred into sample cartridges and loaded into the carousel and submitted to a TDxFLx® instrument for automated analysis. The assay was calibrated using a calibration curve with 6 calibrators (A-F; 0.0, 12.5, 25.0, 50.0, 100,0, 150.0 µg/ml). An acceptable VPA assay calibration curve should meet the following criteria: Polarization Error (PERR)-2.00 to +2.00 for all calibration and root mean squared error (RMSE) less than or equal to 1.00. The calibrators and three controls (low; 37.5 µg/ml, medium; 75.0 µg/ml, and high; 125.0 µg/ml) provided by the manufacturer were tested in duplicate, the same as patient samples and according to the manufacturer's instructions.

***Pharmacokinetic and statistical analyses***

Apparent CL /F were calculated for each patient by using the following equation:

clF(L/hr)=VPA dose mg/[Cpss( mg/L)×τ]

Where CL is the total body clearance of drug, F is the oral bioavailability, Cp_ss_ is reflected trough concentrations and 𝜏 is dosing interval. Just as concentration reflected Cp_ss,_ similarly, calculated CL/F may represent overestimates of the actual values. All data were entered to a database and correlation between groups were analyzed by using the Pearson correlation test and by making comparison between two groups; more than two groups were analyzed by the use of two independent sample T-test and one-way ANOVA test, respectively.(SPSS software for windows, version 11.5 , USA). *P* value less than 0.05 was consider significant.

## Results

***Characteristics of the study populations***

The study population consisted of 47 patients (24 epileptic and 23 manic). Demographic and PK data, medication details and laboratory tests, are summarized in Table 1.

**Table 1:** Characteristics of the study population

Characteristics	*P* value
a. Demographic data	
Patients (n)	47
Age (yr)^ 1^	30.43±12.2
Total Body Weight (kg)^ 1^	64.47±7.57
Male/ Female ratio	0.74
b. Pharmacokinetic data	
Total plasma VPA concentration (mg/l)^ 1 ^	54.51±23.74
VPA CL/F ^2^ (L/hr)^ 1^	0.65±0.55
c. Medication details	
VPA dosage (mg/kg/day)^ 1^	8.93±2.2
Concurrent medication^3^	
Lithium	21%
Antipsychotic therapy	78%
d. Laboratory tests^1^	
AST	22.21±5.95 (U/L)
ALT	24.77±6.74 (U/L)
SCr	0.88±0.13 (mg/dl)
BUN	18.82±4.36 (mg/dl)
FBS	96±4.72 (mg/dl)
WBC	8.33×10^3^/ml ±1.5
RBC	4.45×10^6^/ml±0.52
Platlates	231×10^3^/ml ±53.44

1-Mean±SD, 2- Valproic acid clearance/bioavailability, 3-Percent of patients on concurrent therapy

**Table 2:** Relationship between valporic acid (VPA) apparent clearance and patient age, total body weight and VPA dose

Parameter	Pearson correlation		*P* value^1^
Patients age (yr) and CL/F^2^ (L/hr) Patients TBW^3^ (kg) and CL/F^2^ (L/hr) VPA dose (mg/ day) and CL/F^2^(L/hr)	0.01 -0.13 0.04	0.91 0.35 0.74	

1-Pearson correlation test, 2-Clearance/bioavailability, 3-total body weight

**Table 3:** Comparison of apparent clearance values between patients in different age and body weight groups

	TBW^1^ (kg) group 1 (52-62)	TBW^1^ (kg) group 2 (62-72)	TBW^1^ (kg) group 3 (72-82)	*P* value^2^
Patients (n) Percent (%) CL/F^3^ (L/hr)	20 42.55 0.76±0.17^4^	19 40.42 0.38±0.08^4^	8 17.02 0.12±0.04^4^	0.792
	Age (yr) group 1 (15-30)	Age (yr) group 2 (30-45)	Age (yr) group 3 (45-60)	*P *value^2^
Patients (n) Percent (%) CL/F^3^ (L/hr)	31 65.95 0.6±0.1^4^	14 29.78 0.42±0.11^4^	2 4.25 0.73±0.51^4^	0.573

1-Total body weight, 2-One-way ANOVA**, **3-Clearance/bioavailability, 4-Mean ± SD

***Relationship between patient age, total body weight, VPA dosage and VPA apparent clearance***

Relationship between VPA CL/F and patient’s age, total body weight and VPA dose were analyzed and no significant correlations were found. Results are presented in Table 2. 

The patients were classified into three age groups [15-25 (yr), 25-35 (yr), 35-45 (yr)] and three TBW groups [50-60 (kg), 60-70 (kg), 70-80 (kg)]. The results of VPA CL/F were compared between these three age groups and TBW groups. Results showed no significant differences in VPA Cl/F values between either age groups or between TBW groups. Details are presented in Table 3.

***Influence of gender on VPA CL/F values***

VPA CL/F values between male and female group of patients were compared and no significant difference between these two groups was noted. Results are shown in Table 4.

***Relationship between VPA dosage and total trough plasma concentration***

**Figure 1:**
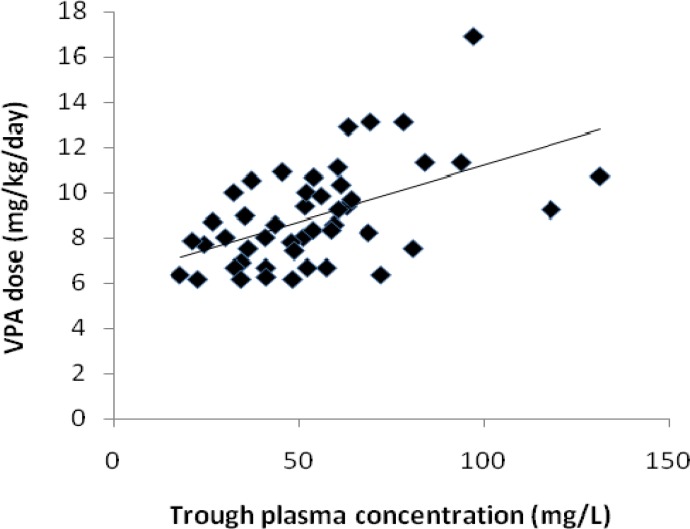
Relationship between valporic acid (VPA) daily dose and total plasma trough concentration

Significant correlation between VPA dose and total trough plasma concentration was found. Results are presented in Table 5 and Figure 1.

**Table 4:** VPA CL/F values in male and female groups and comparison between them

	Male group	Female group	*P* value^1^
Patients (n) VPA CL/F^2 ^(L/h)	20 0.56 ± 0.16^3^	27 0.72 ± 0.71^3^	0.324

1-Two independent sample T test, 2-valproic acid clearance/bioavailability, 3-Mean ± SD

**Table 5:** Relationship between VPA daily dose and total plasma trough concentration

Parameter	Pearson correlation		*P* value^1^
VPA dose(mg/kg/day)and total trough concentration (mg/l)		0.52	0.001

1-Pearson correlation test

## Discussion

Since VPA PK profile may be affected by various factors including VPA dose, patients characteristic, drug-drug interaction and change in its protein binding, this drug have highly inter-patient variable PK (4, 5). In the present study we have evaluated the influence of VPA dose (mg/day) and demographic characteristics on VPA CL/F. The results of our study indicated that VPA CL/F was not affected by patient’s age, TBW, gender and VPA dose. The primary aim of this study was to define pharmacokinetic model which could estimate VPA clearance and determine the influence of covariate factors, in adult Iranian population but since in primary screenings it was found that there is no correlation between VPA clearance and these factors, constructing VPA PK model was not feasible.

 VPA is highly protein bound (90-95%) drug and has low hepatic extraction ratio. The concentration-dependent protein binding of VPA causes nonlinear pharmacokinetics for this drug. Since VPA is eliminated almost completely by means of hepatic metabolism and only the unbound fraction of the drug is available for metabolic transformation, this nonlinear relationship may be explained by the increase in VPA clearance; a fact which would be expected when free VPA concentration increases as a consequence of saturable protein binding (3, 6, 7). In agreement, Herngren and Nergardh showed the protein binding of VPA in plasma was concentration dependent (8). Battino *et al* reported that clearance of VPA positively correlates with unbound concentration (9). Consequently, when using high dose of VPA, its total CL/F is expected to depend on administered daily dose. This is consistent with the results of the Vucicevic *et al* study that showed VPA CL/F increases significantly when VPA dose is more than 1000 mg/day (3). These results are in agreement with other studies investigating the effect of VPA dose on CL/F. In our study patients received various doses of VPA (mean=8.93 mg/kg/day). Since the doses administrated to these patients were low, therefore saturation of protein binding was not expected and the PK of VPA assumed linear. Since in linear PK, there is no correlation between VPA dose and clearance, therefore as it is expected in this study there was no correlation between dose and clearance. This result is in accordance with the results of Bondareva study that reported no relationship between VPA dose and CL/F due to saturation of its protein binding (10). Therefore the relationship between VPA dose and its clearance may be associated with the TDM data. It means that VPA dose may be assign as a covariate in PK models but without TDM data it may be a false covariate. For detailed evaluation it is suggested that VPA dose should be selected in extended ranges.

Although the effect of age on VPA clearance have been frequently studied, most of them have focused on evaluating elderly or children population (9, 11-14). Battino *et al* reported clearance of VPA is strongly age-dependent in pediatric patient and is low in neonates at the end of first postnatal month, and progressively decreases from 2 months to 14 years old (9). Birnbaum *et al* investigated population pharmacokinetics of VPA in elderly patients and reported that CL/F was not affected by age (17). Stephen reported total VPA clearance is similar in young and elderly patients (14). Demographic data (age and TBW) are assumed as covariate factor in some PK models whereas other ones report that VPA CL/F have not been affected by patient's age or weight (3, 15, 16) ,therefore the results are contradictory and imply no conclusive evidence. The results of our study showed that there is no relationship between patient’s age or TBW and VPA CL/F in adult Iranian patients. This is consistent with study reported that VPA CL/F was not affected by neither age nor weight in elderly nursing home residents (17). In contrast, the previous study on VPA PK have shown that CL/F of VPA is strongly age-dependent in pediatric patients. Yukawa *et al* in the other population PK study investigating the effect of covariate factors on VPA clearance (age: 0.3- 54.8 years) reported that VPA relative clearance is in the highest value in the very young and decreases in weight-related fashion in children, with minimal changes observed in adult (16). Jankovic and Milovanovic indicated that the result of PK model showing the CL of VPA increased linearly with TBW and patients age (15). Other studies indicate that VPA CL/F increases with patient's weight. So for detailed evaluation it is suggested to design a larger sample size study with varied and extended age and weight ranges of patients. 

The results of our study also indicate that, there was no gender differences in CL/F and this is in agreement with other studies (13). In contrast in population study Birnbaum *et al* showed the clearance in female patients have been approximately less than that in male patients and in elderly home residents CL/F was 27% lower in female (17).

In this study, it is also indicated that there is significant correlation between VPA dose and its total plasma trough concentration. It is also mentioned that in our study only total (free+bound) concentration were determined whereas it was mentioned that there are significant differences between total and unbound VPA pharmacokinetics parameter (12). Herngren and Nergardh also reported poor correlation between dose, plasma concentration and effect of VPA is contributed to substantial differences between pk of free and total VPA (8). 

Significant variability has been found in the VPA dose- plasma concentration relationship. Battino *et al* indicated very poor correlation between plasma concentration and dose (9). This can be attributed to interindividual differences in the drug clearance.

VPA concentration were found to correlate with the clinical response (3). In contrast some other studies reported there was no direct correlation between efficacy and plasma VPA concentrations (5, 9, 14). Gidal *et al*’s study also showed significant correlation between VPA dose and total and unbound plasma concentration (4).

In addition, although in our study statistically significant correlation existed between VPA dose and total plasma concentration, significant inter-patient variability still remains even under optimal TDM condition.

## Conclusion

Our study showed PK of VPA was not affected by age, sex, TBW and VPA dose. However for detailed results and construction of VPA PK model in Iranian patients, it is necessary to evaluate VPA PK in a larger sample size with different VPA doses, age and TBW ranges. 
